# Surface Modification of Polyester-Fabric with Hydrogels and Silver Nanoparticles: Photochemical Versus Gamma Irradiation Methods

**DOI:** 10.3390/ma12203284

**Published:** 2019-10-10

**Authors:** Kathleen A. Montoya-Villegas, Alejandro Ramírez-Jiménez, Ángel Licea-Claverie, Sergio Pérez-Sicairos, Emilio Bucio, Johanna Bernáldez-Sarabia, Alexei F. Licea-Navarro

**Affiliations:** 1 Centro de Graduados e Investigación en Química, Tecnológico Nacional de México/Instituto Tecnológico de Tijuana, Tijuana 22000, Mexico; kathleen.montoya@tectijuana.edu.mx (K.A.M.-V.); alejandro.ramirezj@tectijuana.edu.mx (A.R.-J.); sperez@tectijuana.mx (S.P.-S.); 2 CONACyT-Centro de Graduados e Investigación en Química, Tecnológico Nacional de México/Instituto Tecnológico de Tijuana, Tijuana 22000, Mexico; 3 Instituto de Ciencias Nucleares, Universidad Nacional Autónoma de México, Circuito Exterior, Ciudad Universitaria, Ciudad de Mexico 04510, Mexico; ebucio@nucleares.unam.mx; 4 Departamento de Innovación Biomédica, Centro de Investigación Científica y Educación Superior de Ensenada, Ensenada 22860, Mexico; jbernald@cicese.edu.mx (J.B.-S.); alicea@cicese.mx (A.F.L.-N.)

**Keywords:** graft polymerization, surface modification, hydrogels, gamma irradiation, silver nanoparticles, antibacterial activity

## Abstract

A Gamma irradiation and photochemical crosslinking/grafting of poly(2-hydroxyethyl methacrylate) (PHEMA) and poly(2-hydroxyethyl methacrylate-co-poly(ethylene glycol) methacrylate) (poly(HEMA-co-PEGMA)) hydrogels onto polyethyleneterephtalate fabric (PET) surfaces were evaluated, in order to obtain a hydrophilic homogeneous coating onto PET fabrics. The materials were characterized by FTIR-ATR, SEM, EDS, and thermal analysis. Furthermore, silver nanoparticles (AgNPs) were loaded by in situ reduction of AgNO_3_, and its antibacterial activity against *Staphylococcus aureus* and *Escherichia coli* was determined. Results showed a ticker coating of hydrogel using gamma radiation and stronger in deep modification of the fibers; however, by the photochemical method, a thin coating with good coverage of PET surface was obtained. The differences in hydrophilicity, thermal properties, and antibacterial activity of the coated fabrics by using both methods were rather small.

## 1. Introduction

Materials for biomedical applications need to fulfill a series of mechanical, physical, chemical, and biological properties to have an adequate performance in contact with a living organism. It is not usual to find a material that meets all the requirements needed for a given application, so the development of new materials by molecular modification of basis materials remains a challenge. Currently there is a renewed interest in textile materials with antimicrobial properties for medical, healthcare, hygiene, and sports applications. Infections associated with medical devices, mainly medical surgical tools and supporting parts, cause at least 1.5–7.2% of post-operational complications. The attachment of microorganisms to the surface of the material is the main problem related to infection and this is the first step in the development of post-operational complications. In this context, chemical modification by grafting is a very good option for biocompatible surface preparation [1,2], however, in this way, the properties of the matrix, like mechanical or chemical resistance, may be slightly affected.

Combination of good mechanical properties of the film supports with the recognized biocompatibility of hydrogel may open novel possibilities. With respect to this, hydrogels obtained from the polymerization of 2-hydroxyethyl methacrylate (HEMA) are well-known in the biomedical and biotechnological fields because of their resemblance to biological tissues, high water content [3,4], low toxicity, high chemical stability to hydrolysis, hydrophilic character [5], and versatility to be copolymerized with functional monomers [6]. Generally, HEMA is thought to be a solvent-like monomer capable of homogenizing hydrophobic and hydrophilic phases [7]; these features mean it is biocompatible and possesses good properties for biomedical uses, for example for drug delivery systems and tissue engineering [8,9,10].

Grafting of polymers onto other polymers by gamma radiation is a suitable technique for surface modification of polymeric materials since this allows to introduce active functional groups on the polymer backbone [11,12,13,14,15]. Graft polymerization combines the features of the matrix, such as chemical and thermal resistance or specific mechanical properties, and introduces functional groups with advantageous new surface properties; this method is applicable for many substrates and combination of monomers and, unlike chemically initiated grafting, it does not require initiators, catalysts, or additives [16]. Moreover, the surface can be covered uniformly, and the polymer is sterilized by the radiation [17].

There are several methods of radiation grafting: (i) The direct (or mutual) grafting method in which the polymeric material is irradiated in contact with the monomer or monomers, although homopolymerization is a collateral effect; (ii) the pre-irradiation method, which involves the irradiation of the polymer matrix in the absence of air and then the grafting is initiated by macroradicals trapped in the irradiated polymer; the main disadvantage is a higher radiation dose than in the direct method, and polymer degradation may occur; and (iii) the pre-irradiation oxidative grafting method that consists of the pre-irradiation of the polymer in the presence of either air or oxygen, so that the macroradicals formed are converted to peroxides and/or hydroperoxides, then when the pre-irradiated polymer is heated, the peroxides decompose to give the macro-radicals, which react with the monomers obtaining a graft polymer [18,19,20]. The grafting yield depends on features of the polymers and monomers such as solubility and radiation stability (degradation and crosslinking) [21].

On the other hand, polyethyleneterephtalate (PET) is a polyester type polymer that has been used in biomedical devices mainly as sutures, cardiovascular grafts, sutures of artificial rings, and in heart valves. PET commercialized as Dacron^TM^ has been successfully used in vascular grafts in long diameter arteries [22,23]; however, the use of PET has been limited due to its low biocompatibility. Therefore, to obtain more biocompatible fabrics, surface modifications have been carried out; for example, Liu and coworkers modified PET surface by grafting of polyamides using UV treatment [24]; Ping and coworkers grafted poly(acrylic acid) by the gamma radiation method, and surfaces were loaded with AgNPs in order to obtain bacteriostatic surfaces [25]; Aubert-Viard and co-workers modified PET and polypropylene by chitosan immobilization, and they loaded iodide/iodate anions or silver cations in order to obtain antibacterial surfaces [26]; Lin and co-workers modified nonwoven PET fabrics by immobilization of antibacterial peptides ε-polylysine and natamicin obtaining excellent antibacterial efficiency [27]; Vesel and co-workers modified PET with heparine in order to obtain more hemocompatible surfaces [28]; Vesel and co-workers carried out another interesting modification introducing –SH functional groups [29] because it is well known that these may be used as anchoring sites for biomolecules. On the other hand; polyesters and polyamides have been treated to improve the binding efficiency of AgNPs due to the poor adhesion between AgNPs and the organic fabrics [30]. PET fabrics treated with silver antimicrobial agents have been extensively studied due to the fact that silver in different forms possesses activity against more than 650 pathogens (bacteria, fungi, and viruses) and has low toxicity towards mammalian cells [31]. Silver antimicrobial agents include silver coating [32], colloidal silver [33], and AgNPs using different methods: Plasma treatment [34,35], corona discharge [36], sonochemical methods [37], and photo reduction of Ag^+^ ions on the surface of other nanoparticles [38]. Despite the different methods used, the challenges remain to modify the surface properties of PET to favor the interactions with the silver and the control of the attachment of AgNPs to avoid their release and the durability of the antimicrobial properties of the silver-modified PET fabrics [39].

The aim of this work was to evaluate the grafting of a hydrophilic gel of poly(2-hydroxyethyl methacrylate) (PHEMA) and of poly (2-hydroxyethyl methacrylate-co-polyethylene glycol methacrylate), poly(HEMA-co-PEGMA), onto PET fabrics using gamma-rays or UV treatment methods in order to obtain hydrophilic/antibacterial surfaces. PET fabrics grafted with poly (HEMA) and poly (HEMA-co-PEGMA) hydrogels were loaded with AgNPs taking advantage from its crosslinked structure, and the antibacterial activity of the modified fabrics against *E. coli* and *S. aureus* was evaluated.

## 2. Materials and Methods

### 2.1. Materials

Polyethyleneterephthalate non-textured fabric (ROGA-0715 Texlon Corp. Torrance, CA, USA), was cut into 9 cm × 9 cm samples for photochemical grafting and into 1 cm × 5 cm for radiation grafting; this was due to the size of the molds used in each reactor. These were then washed with hot water for 24 h in a Soxhlet apparatus, and finally, each sample was dried under vacuum until constant weight. Ethylene glycol dimethacrylate (EGDMA) 98%, 2-hydroxyethyl methacrylate (HEMA), poly (ethylene glycol) methacrylate Mn = 526 (PEGMA), and poly (ethylene glycol) methyl ether methacrylate Mn = 300 (PEGMA300), (Sigma-Aldrich Chemical Co., Toluca, México) were purified by passing through an inhibitor remover column (Aldrich Chemical Co., St. Louis, MO, USA) before use. Sodium borohydride (NaBH_4_) 98%, 1, 2-diphey l-2, 2-dimethoxyethanone 99%, Irgacure^®^ 651, and silver nitrate P. A. ACS 99% (AgNO_3_) (all from Sigma-Aldrich Chemical Co., Toluca, México) were used as received.

### 2.2. Photochemical Crosslinking/Grafting

HEMA (4.8 g, 37 mmol), Irgacure 651 (0.024 g, 0.085 mmol), EGDMA, and PEGMA at different molar ratios (Table 1) were placed into Schlenk flasks; deionized water (80 wt% with respect to HEMA) was used as solvent, and oxygen was displaced by bubbling argon for 6 min. In order to obtain homogeneous solutions, the solutions were sonicated using an ultrasonic bath Branson 2800 (Branson Ultrasonics, Danbury, CT, USA) for 5 min; afterwards, samples of PET fabric were introduced in the Schlenk flasks. Oxygen was thoroughly displaced by using three freeze-thaw cycles using a dry ice/acetone bath and argon flow. Afterwards, the flasks, filled with argon, were sealed, and stored for 12 h inside a refrigerator in order to allow the swelling of the fabric. 

Then, the fabrics were removed from the soaking solution and were placed between two glass plates of 10 cm × 10 cm, sealed with a silicone spacer (1 mm thickness), and the samples were irradiated using lamps of wavelength of 350 nm for 30 min inside a RMR 200 Rayonet Photochemical Chamber Reactor (Palisades Park, NJ, USA) under argon atmosphere. Residual monomers and other compounds were extracted by washing with successive ethanol/water mixtures with increasing ratio of deionized water; finally, the samples were dried under vacuum until constant weight.

### 2.3. Gamma Radiation Grafting

PET fabrics were exposed to ^60^Co γ-source (Gammabeam 651 PT, MDS Nordion, Kanata, ON, Canada) at dose rates of around 10 to 12 kGy h^−1^ and doses between 50 and 70 kGy.

A mixture of 10 mL of HEMA:water or HEMA:PEGMA:water at different volume ratios (Table 2) were placed into a Schlenk flask, argon was then bubbled for 5 min; after that, PET fabrics were soaked with the mixtures and the mixtures were degassed by three freezing cycles with argon flow; afterwards, the flask was sealed and stored inside a refrigerator for 12 h. After that, the fabrics were quickly removed and placed between two glass plates of 2.5 cm × 7.5 cm, sealed with a silicone spacer (0.5 mm thickness), and finally, the samples were irradiated at doses of 50, 60, or 70 kGy. In order to extract the residual monomer and homopolymer formed during the grafting reaction, the samples were soaked four times for 2 h in ethanol-water 25:75 (*V*/*V*, %), followed by ethanol-water 50:50 (*V*/*V*, %) ethanol-water 75:25 (*V*/*V*, %), and ethanol for 12 h each. Afterwards, the samples were dried until constant weight.

The grafting yield (GY) in both methods was calculated using Equation (1):

GY = 100% (W_g_ − W_0_)/W_0_ (1)

where W_0_ and W_g_ represent the weights of the initial and the grafted fabric, respectively.

### 2.4. Characterization of Grafted Hydrogels on PET Fabrics

FTIR-ATR spectra were recorded using a Perkin-Elmer Spectrum 400, FTIR-/FT-NIR spectrometer (Perkin Elmer Cetus Instruments, Norwalk, CT, USA) with 8 or 16 scans between 650 and 4000 cm^−1^. Thermal decomposition of samples was evaluated undo nitrogen flow of 50 mL min^−1^ between 20 and 600 °C at a heating rate of 20 °C min^−1^ using a TGA SDT 2960 Simultaneous DSC-TGA equipment (TA Instruments, New Castle, DE, USA). Glass transition temperature (*Tg*) and melting point (*Tm*) were determined by differential scanning calorimetry (DSC) using a TA-Instrument modulated DSC equipment (DSC 2929) (TA Instruments, New Castle, DE, USA); two heating cycles were recorded in modulation mode under a nitrogen flow of 60 mL min^−1^ at a heating rate of 5 °C min^−1^ with an amplitude of ±0.5 °C over a modulation period of 60 s; in both cycles, the temperature was equilibrated at –10 °C during 5 min before heating to 200 °C for the first cycle, to erase the thermal history, and up to 280 °C for the second one, for measurement. The thickness of the hydrogel thin-film on fabric surface was measured using SEM images of cross-sections. For the cross-section analysis, samples were fractured in liquid nitrogen and fixed on sample holder with a double-sided graphite tape. Analyses were carried-out with a TESCAN VEGA 3 SEM-microscope (Brno, Czech Republic), at an acceleration voltage of 25 kV with a secondary electron detector. Previous to SEM analysis, all samples were sputter-coated with gold for 180 s at 18 mA using a SPI-MODULE sputter coater. The theoretical thickness of coating was 200 Å, according to the manufacturer′s technical information.

Water absorption equilibrium was monitored by immersion of pristine and modified PET fabrics of 2.5 cm × 2.5 cm into distilled water for 96 h. The excess of water on the materials was removed with filter paper and the swollen samples were weighed. The mass-swelling degree in water (Q_water_) of the hydrogel coating was determined using Equation (2).

Q_water_ = 1 + ((W_S_ × ρ_h_)/(W_h_ ∗ ρ_s_)) (2)

W_s_ and W_h_ represent the weights of the adsorbed water and the mass of the dry hydrogel, respectively, where the weight of the fabric without hydrogel was subtracted; and ρ is the density in g/mL. For this work the value of the polymer density PHEMA (ρ = 1.15 g/mL) was taken considering a linear polymer with molecular weight of 20,000 g/mol and ρ = 1.15 g/mL at T = 25 °C, for the polymer PEGMA (ρ = 1.105 g/mL) was taken from a polymer with molecular weight of 300 g/mol at T = 25 °C. For the density of the hydrogel (ρ_h_) the feed composition before crosslinking was taken as a first approximation. The density of water (ρ_s_ = 1.0 g/mL) was used for all calculations. Measurements were performed in triplicate and the average value is reported.

### 2.5. In Situ Synthesis of Silver Nanoparticles

Silver nanoparticles (AgNPs) were synthesized by chemical reduction in situ of AgNO_3_ using NaBH_4_ as reducing agent. Equation (2) shows the chemical reaction:

2AgNO_3_ + 2NaBH_4_ → 2Ag° + H_2_ + B_2_H_6_ + 2NaNO_3_. (3)

Pristine and hydrogel grafted PET fabrics of 1.5 cm × 1.5 cm were swollen in distilled water for five days; after that, samples were soaked in 5 mL of a AgNO_3_ solution (0.005 M); one day later, the pieces of fabrics were transferred into 5 mL of NaBH_4_ solution (0.01 M) at 0 °C. The fabrics were stirred gently for 2 h while cooling with an ice bath; finally, the solution was decanted, and the fabrics were washed with deionized water.

The presence of AgNPs on modified PET fabrics was determined semi-quantitatively by energy dispersive X-ray spectroscopy (EDS), by mapping measurements on surface and cross-sections using a Bruker XFlash Detector 4010 (Berlin, Germany). For surface analysis, samples of 5 mm × 5 mm were cut and fixed on sample holder with a double-sided graphite tape and for cross-section analysis, samples were prepared as described previously for SEM analysis. EDS information was processed using Quantax 200 ESPRIT 1.9 software (Bruker Nano GmbH, Berlin, Germany).

### 2.6. Antibacterial Activity

Bacterial strains were obtained from bacterial culture collection of the Biomedical Innovation Department, at Scientific Research and High Education Center from Ensenada. The Gram (−) strain was *E. coli*, and the Gram (+) strain was *S. aureus*. The antimicrobial activity of hydrogels was performed by modification of the agar disk diffusion method of Collins et al. [40]. Approximately 106 colony-forming units of each bacterium were inoculated on Luria–Bertani (LB) plates. To verify the exponential growth phase of each strain, we measured OD600 values of the bacterial strains over time. The inhibition of the bacterial growth was assessed by triplicate using samples grafted by the photochemical (UV) or gamma irradiation treatments, with or without AgNPs. Silver nanoparticles and silver-free samples were used as a control, and carbencillin (500 μg/mL), a broad-spectrum antibiotic, was used as a positive control. The inhibition zone (mm) was defined as the area in which no bacterial growth was detected. The plates were incubated for 24 h at 37 °C.

## 3. Results and Discussion

### 3.1. Grafting of Hydrogels onto PET-Fabrics

Grafts of HEMA or HEMA/PEGMA were introduced by the photochemical or the direct gamma irradiation methods; grafting percentage was calculated using the Equation (1). Table 1 and Table 2 show the grafting conditions and the hydrogel percentage obtained by the photochemical and the direct gamma irradiation methods, respectively. Results showed that when using the photochemical method, the grafting yield was between 49% and 96%, whereas using the gamma radiation method, it was between 210% and 400%, both related to the weight of the unmodified PET-fabric. The high grafting percentages obtained by gamma irradiation are not good for the application of PET as fabric owing to their increased thickness; even at 50 kGy of irradiation dose, the percentage of grafted hydrogel was too high (285%). These high values may have resulted due to the higher monomer concentrations used, as well as owing to the high radiochemical yield of the monomers and the solvent used. One option to tailor down the grafting percentage could be the use of a mold with a spacer of adequate thickness or the use of a modified grafting method: The so-called oxidative pre-irradiation method previously described [41].

An important step in the synthesis of hydrogels on the PET fabric is the initiation of the polymerization. In this stage, with the photochemical method, free radicals are generated by UV radiation evolving from the photoinitiator (Irgacure ^®^ 651), since this dissociates after the absorption of photons, generating benzoyl and dimethoxybenzoyl radicals, the latter tending to form a methyl radical; both benzoyl radicals and methyl radicals are involved in the polymerization reaction, however the benzoyl radical is more stable than the methyl radical [42]. 

**Scheme 1:**
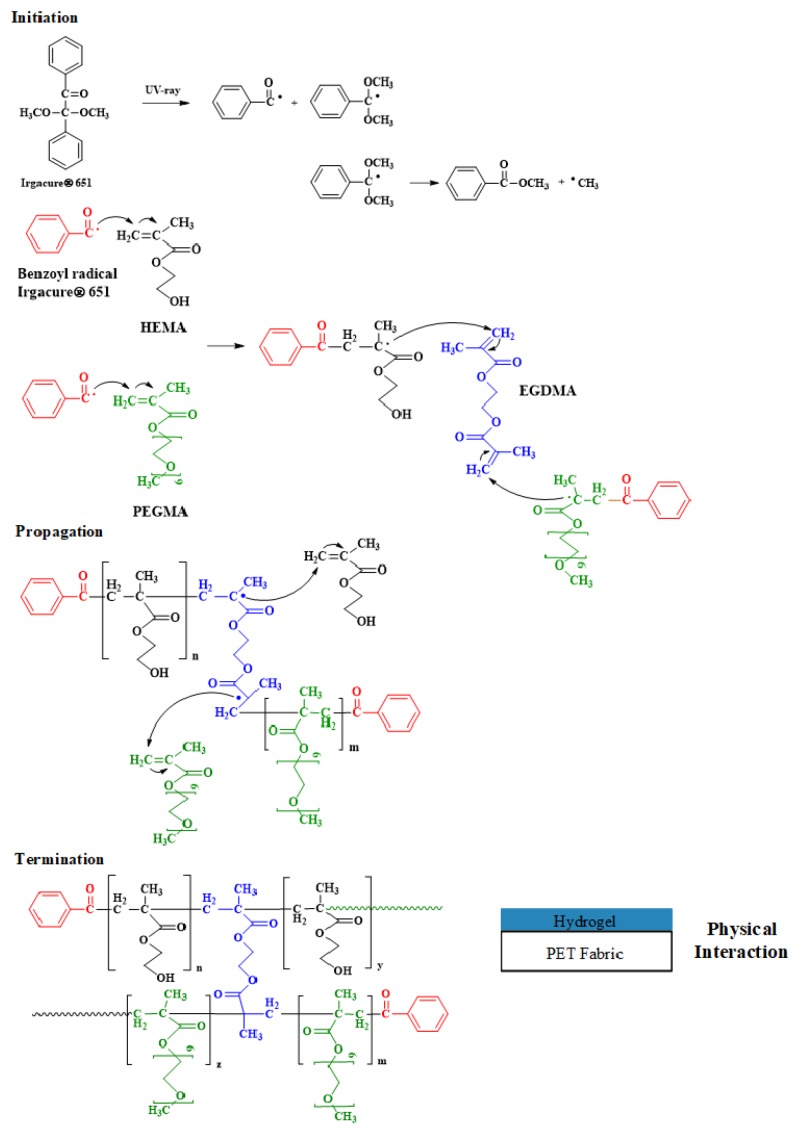
Mechanism of photochemical cross-linking of 2-hydroxyethyl methacrylate (HEMA) and poly (ethylene glycol) methacrylate (PEGMA) onto PET fabric.

**Scheme 2:**
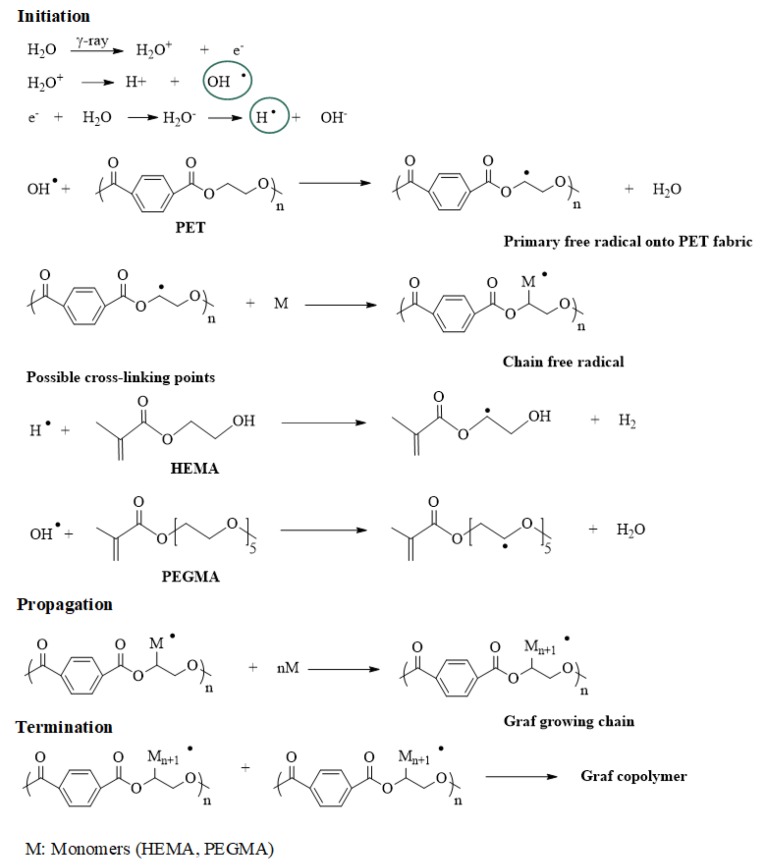
Possible mechanism of grafting HEMA and PEGMA onto PET fabric by γ-ray induced graft copolymerization.

Scheme 1 shows the reaction mechanism for the hydrogels cross-linked with EGDMA, initiated by the photochemical dissociation of the photoinitiator. In the direct gamma irradiation method, the PET backbone can produce two types of free radicals under γ-ray radiation, but –[CH_2__ĊHOOCC_6_H_4_COO]– is the predominant free radical [25,43]. The main procedure possibly involved in the graft copolymerization induced by γ-rays can be described as shown in Scheme 2.

The reactions were carried out in aqueous medium to favor a rapid formation of free radicals on the reagents used, where the reaction is initiated by the formation of free radicals by the radiolysis of deionized water, after the formation of radicals, the coupling of the different species of radicals is favored, forming grafts of polymer networks attached to the PET fabric. Please note that in this case, EGDMA crosslinker was not added.

#### 3.1.1. Characterization of Hydrogel-Grafted PET-Fabrics

##### Water Absorption

Table 1 and Table 2 (last row) show the results of water absorption at the equilibrium for PET fabrics coated with hydrogel by the photochemical method and by gamma irradiation. All samples absorbed water in relatively high values (roughly 2 times their weight). It can be recognized that increased crosslinker content resulted in a lower water absorption, while increasing the PEGMA content resulted in higher water absorption for the hydrogel coatings prepared by the photochemical method (Table 1). In the case of hydrogel coatings prepared by gamma irradiation, the effect of increased water absorption due to the increased PEGMA content was also observed, however the sample prepared without PEGMA also showed a relatively high water absorption (Table 2). Since the crosslinking in the latter case is induced by the gamma rays (no chemical crosslinker added), it is possible that coatings without PEGMA resulted in lower crosslinking degree (higher water absorption). The effect of the coating thickness on water absorption was eliminated by subtracting the PET fabric weight in each case, see experimental section Equation (2).

##### FTIR-ATR

**Figure 1:**
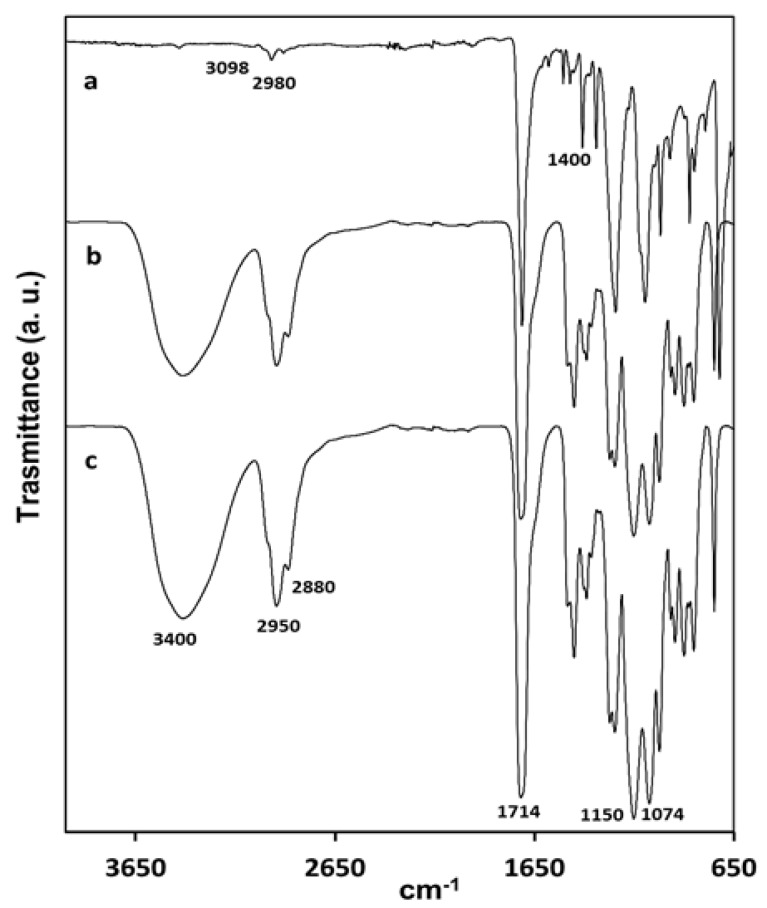
FTIR-ATR spectra of (**a**) unmodified PET, (**b**) UV PET_95:5:0.5_ 67% hydrogel, and (**c**) γ60 PET_90:10:50_ 400% hydrogel.

FTIR-ATR of modified fabrics shows bands centered at 3400 cm^−1^ corresponding to the hydroxyl groups from HEMA; this band was not observed in the pristine PET-fabric spectrum. A very strong band among 1713–1722 cm^−1^ corresponding to asymmetric vibrations of carbonyl groups and others around 1240 and 1095 cm^−1^ corresponding to C-O vibrations from ether groups were observed (Figure 1).

The bands due to the double bond from methacrylate groups were not observed, confirming the polymerization of the monomers included in the synthesis process.

##### Thermal Stability

**Figure 2:**
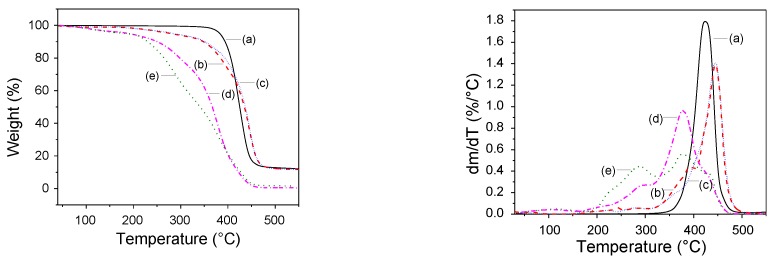
Weight loss and 1st derivate curves of (**a**) unmodified PET, (**b**) UV PET_95:5:0.5_ (67% hydrogel), (**c**) UV PET_100:0:0.5_ (96% hydrogel), (**d**) UV poly(HEMA-co-PEGMA) hydrogel, and (**e**) UV PHEMA hydrogel.

**Figure 3:**
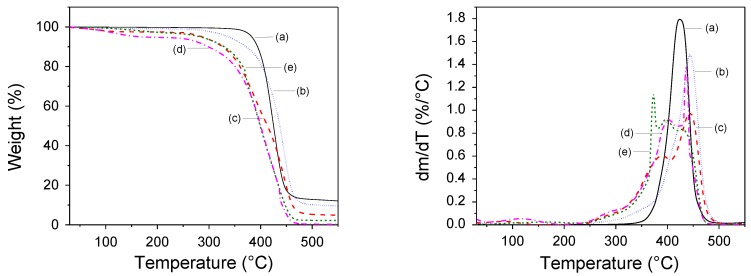
TGA curves of (**a**) unmodified PET, (**b**) γ60 PET_100:0:50_ (257% hydrogel), (**c**) γ60 PHEMA hydrogel, (**d**) γ60 PET_90:10:50_ (400% hydrogel), and (**e**) γ60 poly (HEMA-co-PEGMA) hydrogel.

The thermal stability was measured by TGA; unmodified PET showed a one-step decomposition at an average temperature of 424 °C (Figure 2a), whereas pure hydrogel of PHEMA obtained as a by-product from UV modification showed a loss of volatile compounds at 120 °C (<5%) and thermal decompositions at 285, 374, and 434 °C average temperatures (Figure 2e). Pure PHEMA hydrogel obtained by gamma irradiation showed similar steps of weight loss at average temperatures of 118, 286, 402, and 436 °C (Figure 3c); however, the percentages of weight loss were different. These differences in thermal decomposition may be due to crosslinking induced by gamma radiation forming C-C bonds increased the thermal stability of the hydrogel [21]. PHEMA decomposition occurred via a complex depolymerization mechanism [44]; therefore fabrics grafted with PHEMA by the photochemical method showed three decomposition steps similar to its hydrogel at 278, 360, and 445 °C (Figure 2b), whereas PET modified by gamma irradiation also showed three decomposition steps at 249, 311, and 444 °C (Figure 3b).

These results allowed us to confirm the grafting of PHEMA onto PET fabrics. On the other hand, when poly (HEMA-co-PEGMA) was grafted, small differences in thermal stability were observed; pure hydrogel from the photochemical method showed the same values of thermal decompositions at 287, 378, and 440 °C but with different percentages of weight loss (Figure 2d), whereas pure poly (HEMA-co-PEGMA) hydrogel, obtained by gamma irradiation (Figure 3e), showed decomposition steps at 286, 376, 398, and 432 °C. PET modified with poly (HEMA-co-PEGMA) by the photochemical method showed three steps of decomposition at 242, 389, and 446 °C and PET modified by gamma irradiation also showed three steps of decomposition at 247, 389, and 445 °C. Results confirmed that the hydrogel were attached to PET fabrics.

##### DSC Analysis

**Figure 4:**
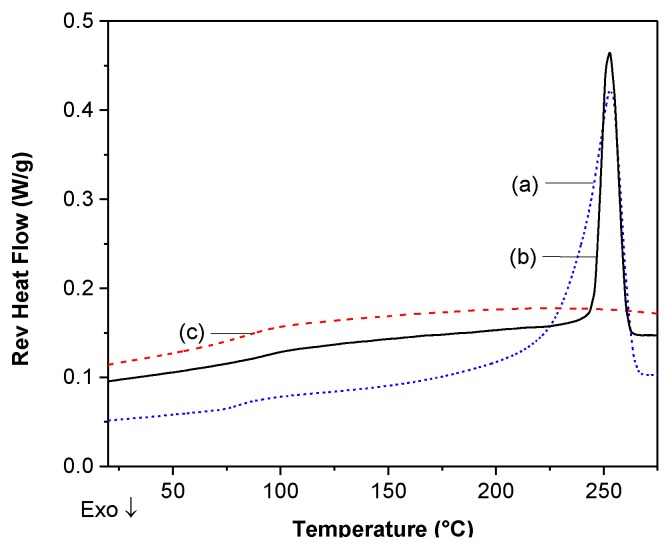
Differential scanning calorimetry (DSC) curves of (**a**) PET, (**b**) UV PET_95:5:0.25_ 96% hydrogel, and (**c**) UV PHEMA hydrogel.

**Figure 5:**
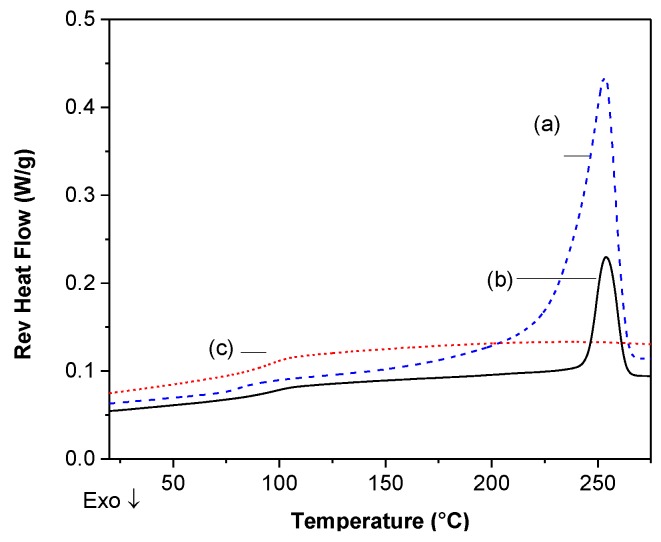
DSC curves of (**a**) PET, (**b**) γ60 PET_90:10:30_ 400% hydrogel, and (**c**) γ60 poly(HEMA-co-PEGMA) hydrogel.

PET is a semi-crystalline polymer with a *Tg* at 80 °C and a *Tm* at 253 °C (Figure 4a); fabrics modified with PHEMA or poly(HEMA-co-PEGMA) by photochemical (UV) method and gamma irradiation showed small variations, which are highlighted in Table 3 and Table 4, respectively. Pure hydrogels are amorphous polymers; the one obtained by UV treatment with 5% of PEGMA showed a *Tg* at 83 °C (Figure 4c), whereas the one obtained by gamma irradiation with 10% of PEGMA showed a *Tg* at 96 °C (Figure 5c). The modified fabrics showed different *Tg* values; in Table 3 and Table 4, we can observe that the higher the PEGMA percentage, the lower the *Tg* and by UV treatment, the higher the EGDMA (crosslinker) content, the higher the *Tg* value.

On the other hand, the higher the gamma radiation dose at a given HEMA:PEGMA ratio, the higher the grafting percentage and the higher the *Tg* value; this as a result of a higher crosslinking effect. In the case of the melting temperature, it was observed that the *Tm* was maintained between 253 and 255 °C, which indicated that the crystalline regions were not affected by the grafting methods; therefore, the grafting was mainly performed in the amorphous regions of PET, independently of the method used.

##### SEM

**Figure 6:**
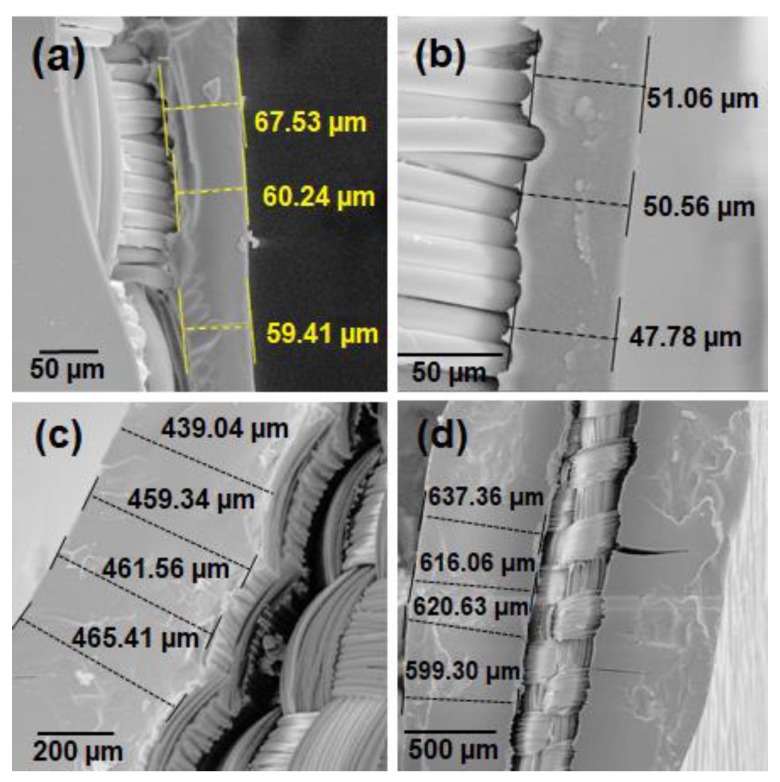
SEM images of (**a**) UV PET_100:0:0.5_, (**b**) UV PET_95:5:0.5_, (**c**) γ60 PET_100:0:50_, and (**d**) γ60 PET_90:10:30_.

Cross-section of PET samples grafted with PHEMA and poly(HEMA-co-PEGMA) using UV method and gamma irradiation were analyzed by SEM and their thicknesses were measured. Samples modified by using the UV method with PHEMA showed a hydrogel cover of roughly 50 μm and the sample modified with poly(HEMA-co-PEGMA) (95:5%) using the same method had a cover of roughly 60 μm (Figure 6a,b). Samples grafted using gamma irradiation with PHEMA showed a thick cover of around 450 µm and of 600 μm for a sample with poly(HEMA-co-PEGMA) (90:10%) (Figure 6c,d). 

**Figure 7:**
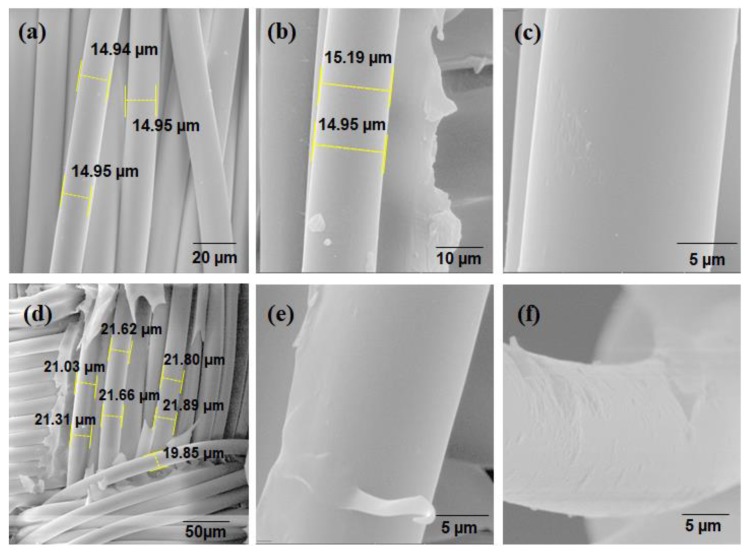
SEM images of fiber PET-fabric: (**a**) Unmodified PET, (**b**) and (**c**) UV PET_95:5:0.5_, (**d**) and (**e**) γ60 PET_100:0:50_, (**f**) γ60 PET_90:10:30_.

Diameters of individual fabric fibers were also measured obtaining mean thickness of roughly 15.0 μm for pristine fibers and of 15.2 μm and 15.0 μm for samples modified by UV method with PHEMA and poly(HEMA-co-PEGMA) (95:5%) respectively (Figure 7), these constant diameters indicated that there was only a surface grafting of the hydrogel. On the other side, by gamma irradiation, an increase in thickness of fibers was observed; for example, the single fibers of the PET sample grafted with PHEMA had diameters around 21 μm; these results indicated that grafting was also performed inside of the fibers [41]. Also, a clear change on fabric fibers surface was observed (Figure 7e) when gamma irradiation was used.

### 3.2. Imparting PET-Fabrics Antibacterial Properties

#### 3.2.1. Incorporation of Silver Nanoparticles

**Figure 8:**
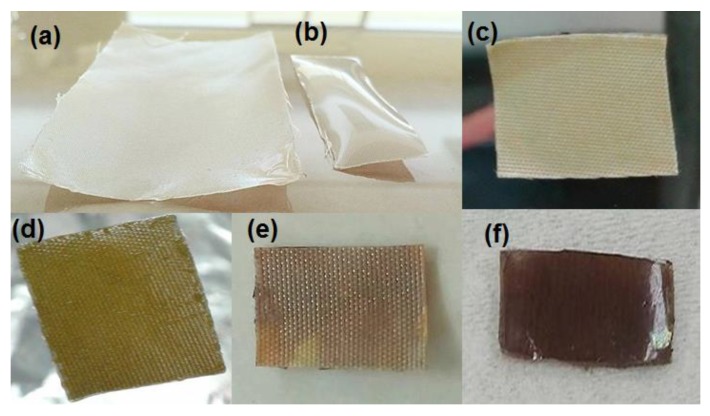
Images of PET fabrics grafted with hydrogels without silver nanoparticles (AgNPs) by (**a**) the photochemical method, (**b**) the gamma irradiation method; and photos of both sides of PET fabrics grafted with hydrogel and AgNPs by method: (**c**,**d**) Photochemical, back and front view, respectively, and (**e**,**f**) gamma irradiation, back and front view, respectively.

The in situ synthesis of AgNPs favors the interaction between the NPs and the hydrogel obtained on PET fabrics surface. As described above, immobilization of Ag^+^ was carried out within the hydrogel and the PET fibers because silver ions from AgNO_3_ solution have affinity toward R-COOH and R-OH groups [45]. The silver ions reduction reaction was carried out using NaBH_4_, this reduction was optically evident since this was accompanied by a color change in the modified fabrics. In Figure 8a,b, the PET fabric with coatings obtained by the photochemical and the gamma irradiation methods, respectively, are observed; white transparent coatings were observed for both samples, however the sample obtained by gamma irradiation presented a thicker coating than the one obtained by the photochemical method. On the other hand, the fabrics modified with AgNPs changed their color. Figure 8c,d shows the back and front of the fabric without hydrogel and with hydrogel obtained by the photochemical method. A yellow color on the front of the fabric is clearly observed, indicating the presence of dispersed AgNPs. Figure 8e,f shows the back and front of the fabric without hydrogel and with hydrogel obtained by gamma radiation. The front cover is brown due to the fact that this side of the fabric has a thick layer of hydrogel, which provides the ability to trap more AgNPs allowing the agglomeration of these within the hydrogel cover. The back of the fabric shows also brownish translucent color from the AgNPs accumulated on the front side.

**Figure 9:**
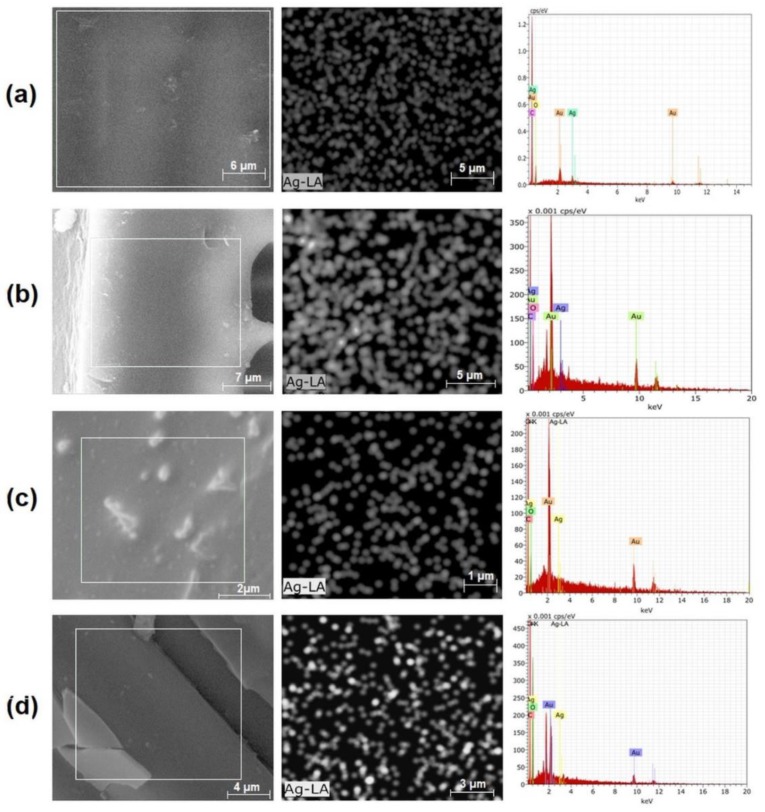
SEM images, EDS mapping of silver, and EDS spectrum from PET fabric modified with hydrogel and AgNPs: (**a**) surface of UV PET_95:5:0.5_, (**b**) cross-section of UV PET_100:0:0.5_, (**c**) surface of γ60 PET_90:10:30_, and (**d**) cross-section of γ60 PET_100:0:50_.

Figure 9 shows the images obtained by SEM along with mapping of silver and elemental analysis (spectrum) from EDS of PET fabrics loaded with AgNPs. From the SEM images, it is not clear where the AgNPs are located; some clustering that can be observed do not correspond with the mapping of silver, which is distributed along the whole sample, both in the surface and also in the cross-section. In Figure 9a,b, some examples of hydrogel-coated PET samples by UV-method (surface and cross-section) are shown. The apparent size of the silver mapping spots do not represent a true size. The same can be concluded from Figure 9c,d, which shows some examples of hydrogel-coated PET samples by the γ60–method. The distribution of AgNPs on the surface of coatings obtained by both the photochemical and the gamma radiation methods were similar; meanwhile, it was evident that the cross-sections demonstrate a high concentration of AgNPs in the PET fabrics prepared using both grafting methods, independently of the thickness of the grafted hydrogel on the surface. The size of the obtained AgNPs was not directly measured on the fabrics, however as an approximation, the same synthetic protocol was repeated but without the presence of the PET fabrics. The obtained AgNPs showed a surface plasmon resonance at a wavelength of 400 nm, which, according to the literature, corresponds to sizes below 20 nm [46].

#### 3.2.2. Antibacterial Test

The antibacterial activity of each sample was determined by measurements of inhibition halo against using two representative bacteria, *S. aureus* and *E. coli* (Table 5). The samples grafted by both methods without AgNPs did not show bacterial inhibition, whereas the samples with AgNPs showed inhibition against the two types of bacteria. By the photochemical method, it was evident that the inhibition was slightly stronger against *E. coli,* which is Gram-negative bacteria, whereas by the gamma radiation method, the samples showed inhibition against *E. coli* in all cases and against *S. aureus* in most of them; the inhibition halo was clearly larger against *E. coli*. Previous studies have suggested that antimicrobial effects of AgNPs may be associated with characteristics of certain bacterial species.

Due to the structural difference in the composition of the membrane of Gram-positive and Gram-negative bacteria, AgNPs have significantly less effect on the growth of Gram-positive bacteria [47]. In our case, the comparison between both types of grafted fabrics (obtained by UV method or by direct gamma irradiation) demonstrated that there was a clear difference in the effectiveness against both types of bacteria when the gamma irradiation method was used for grafting. This may result from the fact that the thicker coating of hydrogel contains a larger amount of AgNPs and, therefore, a higher content on Ag^+^ ions are presumably slowly released from the AgNPs surface. In this investigation, no tests at different contact times were carried out to corroborate the mentioned above; only tests at 24 h were carried out because the biofilm reaches its maturation at this time [48]. The AgNPs size within the samples is another important parameter to be considered in a future work. Besides, according to Taglietti et al., the antimicrobial effect could also be due to a direct contact between bacteria and the AgNPs [39,49].

## 4. Conclusions

Grafting of PHEMA and poly(HEMA-*co*-PEGMA) hydrogels onto the surface of PET fabrics was achieved by two methods: Photochemical crosslinking and gamma irradiation. Photochemical crosslinking used less energy to achieve a smooth and thin (~50 microns) coverage of the PET fabric not affecting the fiber diameter. The hydrogel cover changed the thermal behavior of the PET fabric, increasing its *Tg* with increased crosslinking of the hydrogel and decreasing the *Tg* with the increase in the PEGMA content. The grafted hydrogel was able to contain silver nanoparticles distributed randomly and able to inhibit bacterial growth against *S. aureus* and *E. coli*. Gamma irradiation was used to graft more profoundly PHEMA and poly(HEMA-*co*-PEGMA) into PET fabric, resulting in a thicker coverage of the surface of the fabric (600 microns), and therefore increasing the diameter of the single fibers, with similar impact on thermal properties of PET than the photochemical method; the gamma irradiation modified PET was also able to contain randomly distributed silver nanoparticles. The hydrogel coating containing silver nanoparticles was able to inhibit the growth of *S. aureus* to a similar extent than the photochemical method and inhibit better the growth of *E. coli* than the other method. Hydrogel-coated fabrics by both methods were highly hydrophilic.

## Figures and Tables

**Table 1:** Surface modification of polyethyleneterephtalate (PET) fabric with hydrogel using the photochemical method (λ = 350 nm); water was used as solvent.

Sample	EGDMA (% mol) ^1^	PEGMA (% mol) ^1^	Hydrogel (wt/wt %) ^2^	Q_water_^3^
UV_PET_100_0_0.5_	0.50	–	96	1.7
UV_PET_97_3_0.5_	0.50	3	49	2.0
UV_PET_95_5_0.5_	0.50	5	67	2.1
UV_PET_93_7_0.5_	0.50	7	64	2.1
UV_PET_95_5_0.25_	0.25	5	96	2.3
UV_PET_95_5_1_	1.00	5	85	2.0

^1^ With respect to HEMA monomer; ^2^ calculated using the Equation (1); ^3^ calculated using the Equation (2).

**Table 2:** Surface modification of PET fabric with hydrogel using the direct gamma irradiation method; water was used as solvent.

Sample	Dose (kGy)	PEGMA (% Vol.)	Solvent (% Vol.)	Hydrogel (wt/wt%) ^1^	Q_water_^2^
γ60_PET_100_0_50_	60	–	50	257	2.5
γ60_PET_90_10_50_	60	10	50	400	1.8
γ60_PET_90_10_40_	60	10	40	400	1.8
γ60_PET_90_10_30_	60	10	30	400	1.8
γ60_PET_80_20_50_	60	20	50	213	1.9
γ60_PET_70_30_50_	60	30	50	285	2.9
γ50_PET_90_10_50_	50	10	50	285	1.8
γ70_PET_90_10_50_	70	10	50	285	1.8

^1^ Calculated using the Equation (1); ^2^ calculated using the Equation (2).

**Table 3:** Results by DSC for PET fabrics modified with hydrogel, obtained by the photochemical method.

Sample	HEMA:PEGMA (% mol)	EGDMA (% mol)	Hydrogel (wt/wt %)	T_g_ (°C)	T_m_ (°C)
Unmodified PET fabric	-	-	-	80	253
UV_PET_100_0_0.5_	100:0	0.5	96	79	254
UV_PET_97_3_0.5_	97:3	0.5	49	108	255
UV_PET_95_5_0.5_	95:5	0.5	67	98	253
UV_PET_93_7_0.5_	93:7	0.5	64	58	255
UV_PET_95_5_0.25_	95:5	0.25	96	91	253
UV_PET_95_5_1_	95:5	1	85	110	254

**Table 4:** Results by DSC for PET fabrics modified obtained by the direct gamma irradiation method.

Sample	Dose (kGy)	H:P (v/v %)	Hydrogel (wt/wt%)	T_g_ (°C)	T_m_ (°C)
Unmodified PET fabric	-	-	-	80	253
γ60_PET_100_0_50_	60	100:0	257	95	255
γ60_PET_70_30_50_	60	70:30	400	54	255
γ60_PET_80_20_50_	60	80:20	400	76	255
γ60_PET_90_10_50_	60	90:10	400	91	255
γ60_PET_90_10_40_	60	90:10	213	96	254
γ60_PET_90_10_30_	60	90:10	285	98	254
γ50_PET_90_10_50_	50	90:10	285	88	255
γ70_PET_90_10_50_	70	90:10	285	93	255

**Table 5:** Antibacterial results of modified PET fabrics.

Sample	Inhibition Distance (mm)		Inhibition Distance with Ag NP’s (mm)		Hydrogel (%)
	*S. aureus*	*E. coli*	*S. aureus*	*E. coli*	
UV_PET_100:0:0.5_	0	0	0.83 ± 0.3	1.00 ± 0.0	96
UV_PET_97:3:0.5_	0	0	0.67 ± 0.6	1.33 ± 0.6	49
UV_PET_95:5:0.5_	0	0	0.67 ± 0.6	0.67 ± 0.6	67
UV_PET_93:7:0.5_	0	0	0.83 ± 0.3	1.83 ± 0.3	64
UV_PET_95:5:0.25_	0	0	1.00 ± 0.5	1.33 ± 0.6	96
γ60_PET_100:0:50_	0	0	0	2.0 ± 0.0	257
γ60_PET_90:10:50_	0	0	0.83 ± 0.6	2.33 ± 0.6	400
γ60_PET_80:20:50_	0	0	0	3.67 ± 0.8	213
γ60_PET_70:30:50_	0	0	1.33 ± 1.3	3.33 ± 0.6	285
γ70_PET_90:10:50_	0	0	0.67 ± 0.5	2.17 ± 0.8	285
